# Marked inactivation of *O*^6^-alkylguanine-DNA alkyltransferase activity with protracted temozolomide schedules

**DOI:** 10.1038/sj.bjc.6600827

**Published:** 2003-04-01

**Authors:** A W Tolcher, S L Gerson, L Denis, C Geyer, L A Hammond, A Patnaik, A D Goetz, G Schwartz, T Edwards, L Reyderman, P Statkevich, D L Cutler, E K Rowinsky

**Affiliations:** 1 Institute for Drug Development, Cancer Therapy and Research Center, San Antonio, TX, USA; 2 Department of Medicine, Division of Medical Oncology, University of Texas Health Science Center at San Antonio, USA; 3 Case Western Reserve University, Cleveland OH, USA; 4 Joe Arrington Cancer Center, Lubbock TX, USA; 5 Brooke Army Medical Center, San Antonio TX, USA; 6 Schering-Plough Research Institute, Kenilworth, NJ, USA

## Abstract

Temozolomide, an oral DNA methylator that inactivates the DNA repair enzyme *O*^6^-alkylguanine-DNA alkyltransferase (AGAT), has demonstrated anticancer activity on protracted schedules. Protracted schedules may lead to an ‘autoenhancement’ of temozolomide's inherent cytotoxic potential by cumulative reduction of the cell's capacity for AGAT-mediated DNA repair and resistance. This study was undertaken to characterise AGAT inactivation and regeneration in the peripheral blood mononuclear cells (PBMCs) of patients treated on two protracted temozolomide schedules. *O*^6^-alkyl guanine-DNA alkyltransferase activity was measured in the PBMCs of patients treated on two phase I protracted temozolomide studies. Patients were treated daily for either 7 days every 2 weeks (Schedule A) or 21 days every 4 weeks (Schedule B). The effects of various temozolomide doses (75–175 mg m^−2^), treatment duration (7–21 days), and temozolomide plasma levels on AGAT inactivation and regeneration, as well as the relation between AGAT inactivation and toxicity, were studied. *O*^6^-alkyl guanine-DNA alkyltransferase activity in PBMCs was measured serially in 52 patients. Marked inactivation of AGAT occurred following 7 days of temozolomide treatment, with mean AGAT activity decreasing by 72% (*P*<0.0001). Similarly, mean AGAT activity decreased by 63 and 73% after 14 and 21 days of treatment, respectively (*P*<0.001 for both comparisons). *O*^6^-alkyl guanine-DNA alkyltransferase inactivation was greater after 7 days of treatment with higher doses of temozolomide than lower doses and remained markedly reduced 7 days post-treatment. However, AGAT inactivation following temozolomide treatment for 14 and 21 days was similar at all doses. On the continuous 21-day schedule, AGAT inactivation was significantly greater in patients who experienced severe thrombocytopenia than those who did not (90.3±5.5 *vs* 72.5±16.1%, *P*<0.045). In Conclusion, protracted administration of temozolomide, even at relatively low daily doses, leads to significant and prolonged depletion of AGAT activity, which may enhance the antitumour activity of the agent.

Temozolomide (Temodar, SCH52365, NSC362856), an oral imidazotetrazinone methylating agent, undergoes spontaneous conversion in plasma to its active methylating species, 3-methyl-(triazen-1yl)imidazole-4-carboxamide (MTIC) (Hickman *et al*, 1985; Stevens *et al*, 1987; Tsang *et al*, 1990,^1991^). In both preclinical and clinical investigations, temozolomide has demonstrated markedly schedule-dependent anticancer activity, with more frequent treatment schedules generally producing greater cytotoxicity than less frequent schedules (Stevens *et al*, 1987). In one series of studies, temozolomide's activity against several human tumour xenografts was much greater when the agent was administered daily for 5 days compared to schedules, in which an equivalent total dose was administered every other day or as a single treatment (Stevens *et al*, 1987). In sequential phase I studies of temozolomide, the response rate was also greater with temozolomide administered daily for 5 days every 4 weeks than when an equivalent total dose was administered as a single treatment every 4 weeks (7 of 42 (17%) *vs* 0 of 51 (0%)) (Newlands *et al*, 1992).

The temozolomide dose schedule that has been granted regulatory approval for treating patients with refractory anaplastic astrocytoma is 150–200 mg m^2^ day^−1^ for 5 days every 4 weeks; however, intriguing results have recently been noted with temozolomide administered on protracted low-dose schedules. A 47% response rate was noted in patients with recurrent glioblastoma multiforme who were treated with temozolomide, 75–100 mg m^2^ day^−1^, on a protracted daily schedule for 6–7 weeks, which compares favourably with the response rate in similar patients treated with temozolomide on its approved dose schedule (Newlands *et al*, 1992; Bower *et al*, 1997; Brock *et al*, 1998).

One mechanism that has been proposed to explain the schedule-dependent anticancer activity of temozolomide, as well as the encouraging, although preliminary, results noted with protracted low-dose schedules, is that frequent temozolomide treatment, particularly on protracted schedules, may lead to marked, cumulative, and sustained inactivation of *O*^6^-alkylguanine-DNA alkyltransferase (AGAT). *O*^6^-alkylguanine-DNA alkyltransferase is an ubiquitous DNA repair enzyme that removes naturally occurring DNA damage because of methylating and alkylating lesions at the *O*^*6*^ position of guanine and covalently links these methyl and alkyl groups to an internal cysteine amino acid present within the AGAT protein (Tano *et al*, 1990; Walter *et al*, 2001; Zhou *et al*, 2001). As a consequence of this ‘housekeeping’ function, AGAT confers cellular resistance to chemotherapeutic agents that principally target the *O*^6^ position of guanine, including chloroethylating (e.g. BCNU, and CCNU) and methylating (e.g. procarbazine, streptozotocin, dacarbazine, and temozolomide) agents (Dumenco *et al*, 1989; Schold *et al*, 1989; Tano *et al*, 1990; Jaeckle *et al*, 1998). In both experimental and clinical settings, tumours with high AGAT expression have demonstrated marked resistance to chlorethylating and methylating agents (Dumenco *et al*, 1989; Schold *et al*, 1989; Wu *et al*, 1992; Friedman *et al*, 1998; Jaeckle *et al*, 1998; Esteller *et al*, 2000). Following the covalent transfer of methyl- or alkyl groups to the AGAT protein, AGAT is irreversibly inactivated such that *de novo* synthesis of new AGAT protein is required for recovery of cellular AGAT function (Pegg, 1990; Tano *et al*, 1990). Therefore, administering methylating agents, such as temozolomide, on schedules that result in cumulative and sustained inactivation of AGAT may, in essence, ‘auto-enhance’ their own inherent cytotoxic activity by further reducing the cell's capacity for DNA repair and intrinsic drug resistance (Dolan *et al*, 1990).

Based on the intriguing, albeit preliminary, clinical results reported with protracted, low-dose temozolomide schedules, this pharmacodynamic study was undertaken to characterise AGAT inactivation and regeneration in the peripheral blood mononuclear cells (PBMCs) of patients treated with temozolomide administered daily for either 7 days every 2 weeks or 21 days every 4 weeks.

## MATERIALS AND METHODS

### Patient selection

Patients with histologic or cytologically confirmed solid malignancies refractory to standard therapy, or for whom no standard therapy existed, were entered into one of two concurrent phase I studies of temozolomide. Patient entry criteria also included: age ⩾18 years; life-expectancy of at least 12 weeks; a Eastern Cooperative Oncology Group (ECOG) performance status of 0–2; no prior chemotherapy within 4 weeks (6 weeks for prior mitomycin C or a nitrososurea); adequate haematopoietic (haemoglobin ⩾9 g dl^−1^, absolute neutrophil count (ANC) ⩾1500 *μ*l^−1^, platelet count ⩾100 000 *μ*l^−1^), hepatic (bilirubin< 1.5 mg dl^−1^, aspartate serum transferase (AST) and alanine serum transferase (ALT) ⩽3 times the upper limit of normal, or <5 times the upper limit of normal if the elevation was because of hepatic metastases), and renal (serum creatinine ⩽1.5 mg dl^−1^) functions; measurable or evaluable disease; no prior high-dose chemotherapy requiring haematopoietic stem cell support; and no coexisting medical problem of sufficient severity to limit compliance with the study. The protocol was approved by the institutional review board at each participating centre and patients gave written informed consent for all clinical and research aspects of the study according to federal and institutional guidelines prior treatment.

### Drug administration

Temozolomide capsules were supplied by Schering-Plough Research Institute (Kenilworth, NJ, USA) in 5, 20, 100, and 250-mg strengths. All doses were rounded up to the nearest 5-mg to accommodate capsule strength. Patients were instructed to fast for 2 h prior to dosing and 2 h postdosing. Patients were also instructed to ingest the temozolomide capsules at approximately the same time each treatment day, and not to chew or crush the capsules.

### Treatment schedules

Patients were treated with temozolomide on one of the following two protracted administration schedules:

*Schedule A. Temozolomide administered daily for 7 days repeated every 2 weeks*. Temozolomide capsules were administered to successive cohorts of patients at doses ranging from 50 to 175 mg m^−2^ orally once daily for seven consecutive days repeated every 2 weeks. A course of therapy was defined as 4 weeks.

*Schedule B. Temozolomide daily for 21 days repeated every 4 weeks*. Temozolomide capsules were administered to successive cohorts of patients at doses ranging from 50 to 150 mg m^−2^ orally once daily for 21 consecutive days repeated every 4 weeks. A course of therapy was defined as 4 weeks.

### *O*^6^-alkylguanine-DNA alkyltransferase activity

To measure AGAT activity in PBMCs, 10-ml blood samples were collected into Vacutainer CPT™ tubes (Becton Dickinson, Franklin Lakes, NJ, USA) on day 1 (prior to the first dose), day 8 (24 h after the seventh dose), day 15 (prior to starting second 7-day treatment), and day 22 (24 h after the last dose in course 1) in patients receiving temozolomide on Schedule A. For patients treated with temozolomide on Schedule B, blood sampling was performed prior to treatment on days 1, 15, and 22 (24 h after the last dose) in course 1. Sample processing was performed within 30 min of collection. The CPT™ tubes were centrifuged at room temperature for 25 min at 2800 revolutions per minute (rpm) followed by 10 min at 1900 rpm. The interface containing the PBMCs was resuspended in 40 ml of phosphate-buffered saline (PBS) at 1 mM EDTA, and centrifuged at 1500 **g** for 10 min at 4°C. The cells were immediately resuspended and washed again in 40 ml of PBS at 1 mM EDTA. The PBS was removed and the cell pellet frozen at −80°C in an Eppendorf tube.

The activity of AGAT in PBMCs was measured by sonicating the cells contained in the pellet and quantifying the removal of a [^3^H]methyl adduct from the *O*^6^ position of guanine in a DNA substrate when incubated with cell extracts as described previously (Spiro *et al*, 1999).

### Pharmacokinetic sampling and assay

Blood samples (5 ml) for pharmacokinetic studies were collected into prechilled syringes through an indwelling venous catheter, emptied into prechilled heparinised tubes, and immediately cooled in an ice water bath. Patients treated with temozolomide on Schedule A had blood sampling performed prior to treatment on days 1–7 and 15, as well as 15 and 30 min, and 1, 1.5, 2, 2.5, 3, 4, 6, and 8 h after treatment on days 1 and 15 in course 1. For patients treated with temozolomide on Schedule B, blood sampling was performed prior to treatment on days 1 and 21, as well as 15 and 30 min, and 1, 1.5, 2, 2.5, 3, 4, 6, and 8 h after treatment on days 1 and 21 in course 1.

Within 30 min of collection, the blood samples were centrifuged at 3000 rpm for 10 min at 4°C. Following centrifugation, 2 ml of plasma was immediately transferred to a plastic tube containing 0.10 ml of 8.5% phosphoric acid. The acidified plasma was vortexed, separated into two 1-ml aliquots, and the aliquots were placed into plastic tubes that were frozen immediately at −20°C.

A validated high-performance liquid chromatography (HPLC) method was used to quantify plasma temozolomide concentrations and has been described previously (Kim *et al*, 2001). Briefly, to each 500 *μ*l of plasma, 500 *μ*l (50 *μ*g) of an internal standard (IS) solution (ethazolastone, Schering Plough Research Institute, Kenilworth, NJ, USA) and 1 N HCl (50 *μ*l) were added followed by 5 ml of ethyl acetate. The solution was then vortexed, centrifuged and the organic layer transferred, and the solvent evaporated to dryness. The residue was dissolved in 0.3 ml of mobile phase, transferred to autosampler vial inserts and 20 *μ*l was injected onto the HPLC column. The HPLC system consisted of a Shimdzu LC-9A pump and a Model 3200 absorbance detector (Shimadzu Scientific Instruments, Princeton, NJ, USA) set at 316 nm. Separation was accomplished on a Ultrasphere ODS, 5 *μ*m, 150 × 4.6 mm^2^ column that was preceded by an on-line filter. The mobile phase, which consisted of 0.1% aqueous acetic acid–acetonitrile (90/10, v v^−1^), and pumped at a flow rate of 1.0 ml min^−1^.

A calibration curve was analysed using acidified plasma samples freshly spiked in triplicate. Each sample contained 0.10, 0.20, 0.50, 1.0, 5.0, 10.0, and 20.0 *μ*g ml^−1^ of temozolomide per millilitre and a fixed amount (1.0 *μ*g ml^−1^) of IS. Similarly prepared quality control samples, which had been spiked with temozolomide (0.2, 1.5, and 15.0 *μ*g ml^−1^) and stored frozen (–80°C), were processed and assayed in triplicate. Standard curves were constructed by plotting the ratio of temozolomide peak areas to those of the IS *vs* known plasma concentrations. A linear response between 0.10 and 20.0 *μ*g ml^−1^ was observed (*r*>0.99) for all calibration curves. The coefficient of variation ranged between 0.0 and 3.7%, the bias ranged from –10.0 to 7.5%, and the interassay coefficient of variation (CV%) was no more than 6.9% over analysis period.

### Pharmacokinetic and pharmacodynamic analyses

Individual temozolomide plasma concentration data sets from days 1, 15, and 21 were analysed by noncompartmental methods. Peak concentrations were determined from inspection of individual patient plasma concentration–time curves. Elimination rate constants were estimated by linear regression of the last three data points on the terminal log linear portion of the concentration–time curves. Terminal half-lives (*t*_1/2_) were calculated by dividing 0.693 by the elimination rate constants. Values for area under the concentration *vs* time curve (AUC) were calculated using the linear trapezoidal rule up to the last measurable data point (AUC_0−*t*_), or extrapolated to infinity (AUC_0−∞_). The apparent systemic clearance (CL/*f*) was determined by dividing the dose of temoz-olomide in mg m^−2^) by the AUC. The temozolomide AUC up to the time of PBMC sampling for AGAT activity (AUC_0−*t*_) was estimated by multiplying the AUC_0−∞_ on day 1 and the number of days of continuous temozolomide administration prior to PBMC sampling.

The relations between the percentage AGAT inactivation percentage and pharmacokinetic parameters reflecting exposure were explored. Specific pharmacokinetic parameters that were evaluated included AUC_0−∞_ values on day 1 and total AUC up to the time of PBMC sampling (AUC_0−*t*_), estimated as the product of AUC_0−∞_ and the number of days of treatment up to PBMC sampling × days and *C*_max_ × days) and the percent decrement in AGAT activity from pretreatment values in course 1 were explored. The percentage inactivation of AGAT activity was calculated as follows: 100% × [pretreatment AGAT activity−AGAT activity at sampling]/pretreatment AGAT activity]). Pharmacodynamic relations were fit to a linear model and a maximal effect (*E*_max_) model of drug action, in which the percentage change in AGAT=*E*_max_ × AUC_*γ*0_/AUC_50*γ*_+AUC_*γ*_). The maximal effect (*E*_max_) was fixed at 100%, the AUC_50*γ*_ was the AUC at which the effect is 50% of the maximal effect, and the exponent *γ* was a constant that describes the sigmoidicity of the curve. Both simple and sigmoidal *E*_max_ models were fit to the data by nonlinear least-squares regression. The coefficient of determination (*R*^2^) and the standard errors for the estimated parameters were used as measures of goodness of fit for the pharmacodynamics model. Parameter values were expressed as means and standard deviation values.

The relations between the percent decrement in platelets and neutrophils from baseline over the first two courses and the percent decrement of AGAT activity in the first course were explored. The percent decrements in blood cell counts were calculated as follows: 100% × ([pretreatment counts−nadir counts]/pretreatment counts). The relations between decrements in platelet and neutrophil counts and AGAT were assessed both by a linear model of correlation, and by using an *E*_max_ model.

### Statistical analysis

Mean values for AGAT activity were calculated and compared using either a paired or unpaired Student's *t*-test. Analysis of variance (repeated measures) was used to compare the mean AGAT activity of patients sampled at pretreatment, days 15 and 22. The correlation coefficient (Spearman) was calculated for decrements in AGAT activity and decrements in neutrophil and platelet counts.

## RESULTS

### General

A total of 72 patients were entered onto the two concurrent phase I studies of temozolomide administered on Schedules A and B. For Schedule A, the maximum tolerated dose (MTD) of temozolomide was determined to be 150 mg m^−2^ day^−1^ for 7 days every 2 weeks for both minimally and heavily pretreated patients (Figueroa *et al*, 2000). For Schedule B, the MTDs were determined to be 85 and 100 mg m^−2^ day^−1^ for 21 days every 4 weeks for heavily and minimally pretreated patients, respectively (Denis *et al*, 2000). Severe thrombocytopenia, often associated with severe neutro-penia, was the principal dose-limiting toxicity encountered with both administration schedules. The complete clinical toxicologic and pharmacokinetic profiles of temozolomide on these schedules are being reported separately (Denis *et al*, 2000; Figueroa *et al*, 2000).

### Effects of temozolomide on PBMC AGAT activity

**Figure 1:**
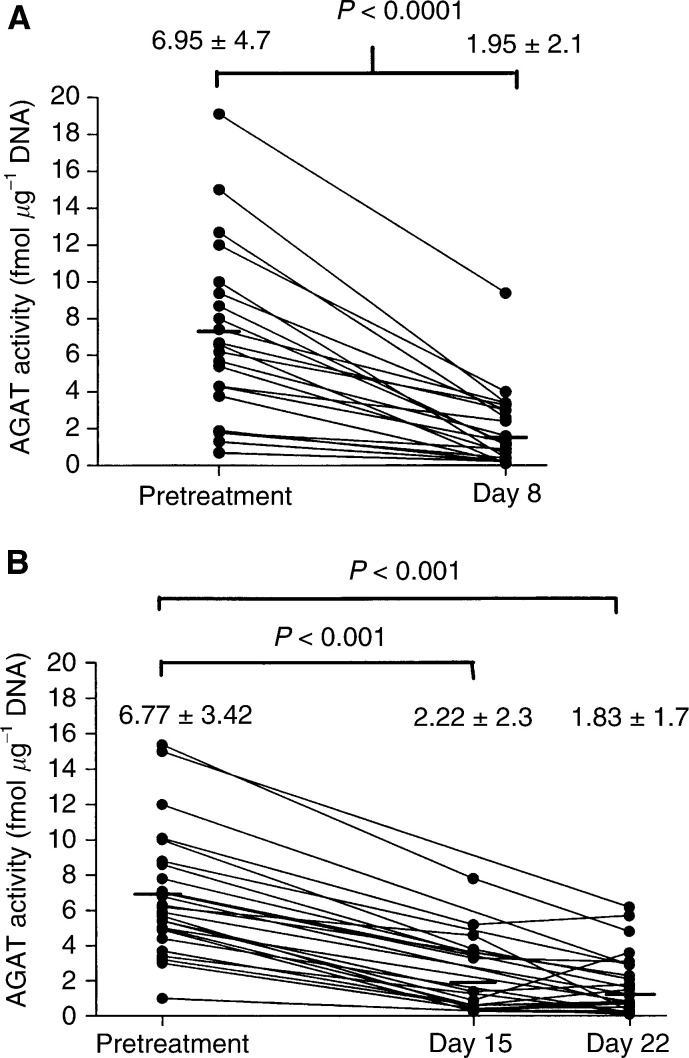
Scatterplots of AGAT activity in PBMCs sampled (**A**), pretreatment and after 7 days of daily temozolomide treatment administered (at doses of 75–175 mg m^−2^ day^−1^) on Schedule A; and (**B**) after 14 and 21 days of temozolomide treatment (at doses of 85–125 mg m^−2^ day^−1^) on Schedule B.

Peripheral blood mononuclear cells were collected serially in 52 of the 72 patients enrolled in the two clinical studies. In all, 20 patients did not have samples collected; 10 who were entered into the clinical studies prior to the protocol amendment for AGAT analysis, and 10 patients who had inadequate samples for analysis. O^6^-alkylguanine-DNA alkyltransferase activity in PBMCs was measured serially in 25 and 27 patients treated with temozolomide on Schedules A and B, respectively. Scatterplots of all assessments of PBMC AGAT activity are displayed in Figure 1A, B

. In patients treated with temozolomide on Schedule A, marked inactivation of AGAT was noted after 7 days of daily treatment, at which time AGAT activity decreased by 72%, on average, from 6.95±4.7 to 1.95±2.11 fmol *μ*g^−1^ DNA (paired *t*-test, *P*<0.0001). Similarly, mean AGAT activity decreased by 63 and 73% from pretreatment levels following 14 and 21 days of temozolomide treatment (Schedule B), respectively (paired *t*-test, *P*<0.001 for both comparisons). In the subsets of patients treated at the MTDs, mean AGAT activity decreased by 80.1±14.1% after 7 days of temozolomide treatment (Schedule A), whereas mean AGAT activity decreased by 74.5±16.4% after 21 days of treatment (Schedule B) (paired *t*-test, *P*<0.0001 for both comparisons).

**Figure 2:**
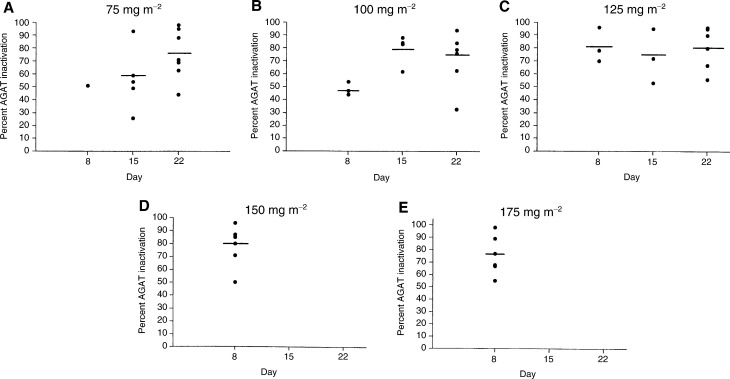
Scatterplots of the percentage values of PBMC AGAT inactivation for Schedules A (day 8 values) and B (day 15 and 22 values) at each temozolomide dose: (**A**) 75 mg m^−2^ day^−1^ (day 8 only) and 85 mg m^−2^ day^−1^ (days 15 and 22); (**B**) 100 mg m^−2^ day^−1^; (**C**) 125 mg m^−2^ day^−1^; (**D**) 150 mg m^−2^ day^−1^; and (**E**) 175 mg m^−2^ day^−1^. Dose-limiting toxicity precluded daily treatment with temozolomide for longer than 7 days at the 150 and 175 mg m^−2^ day^−1^ dose levels.

After 7 days of daily temozolomide treatment, the percentage decrements in AGAT activity were generally greater at the higher dose levels (125, 150, and 175 mg m^−2^ day^−1^) than at the lower dose levels (75 and 100 mg m^−2^ day^−1^), although the small number of patients treated at the lowest dose level were insufficient to provide statistical analysis of the dose-dependence of AGAT inactivation. The percentage decrements in AGAT activity averaged 51±0, 48±5, 81±13, 80±14, and 76±16% following 7 days of treatment with temozolomide doses of 75, 100, 125, 150, and 175 mg m^−2^ day^−1^, respectively. With more protracted temozolomide treatment (14 and 21 days), dose-related effects were no longer apparent and AGAT inactivation was similar at all dose levels. Furthermore, AGAT inactivation following 14 and 21 days of treatment with low doses temozolomide (85 and 100 mg m^−2^ day^−1^) was comparable to that achieved with higher doses (125, 150, and 175 mg m^−2^ day^−1^) administered for 7 days (Figure 2A–E

).

**Figure 3:**
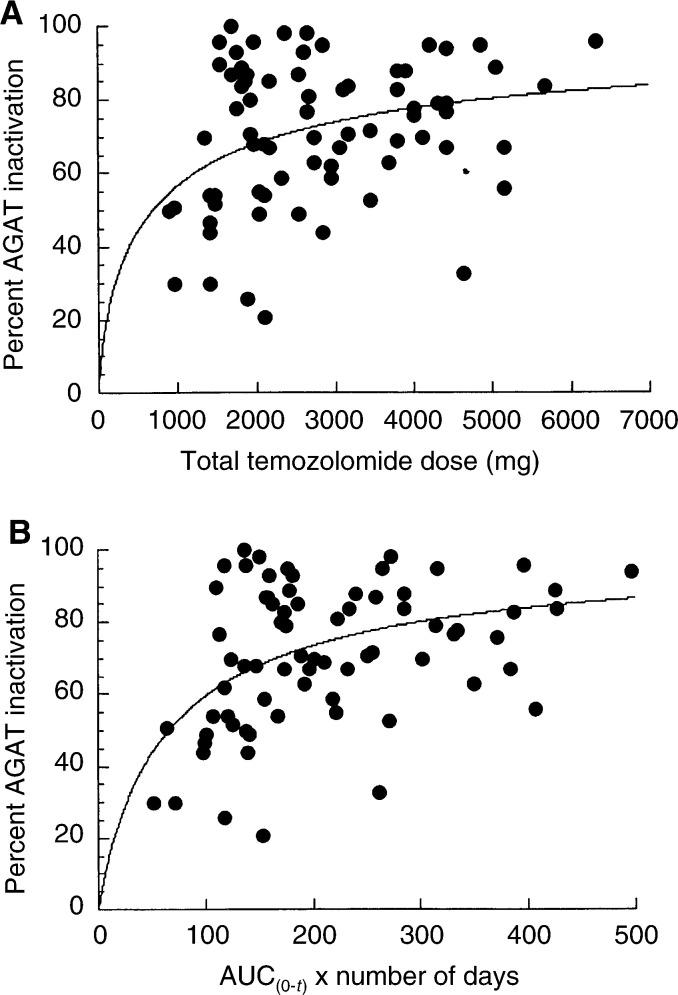
(**A**) Scatterplot of the percentage values of PBMC AGAT inactivation as a function of the total cumulative temozolomide dose administered up to the time of PBMC sampling (*R*^2^=0.102). (**B**) Scatterplot of temozolomide exposure as the product of AUC_0-*t*_ multiplied by total days temozolomide administered prior to PBMC sampling (*R*^2^=0.188).

Since both dose and duration of temozolomide treatment appeared to be related to AGAT inactivation, relations between AGAT inactivation and both total temozolomide dose and AUC_0−*t*_ were sought. A scatterplot of the percentage decrements in AGAT activity in all PBMC samples as a function of the total temozolomide dose (in mg) and plasma AUC administered up to the time of PBMC sampling is displayed in Figure 3

. Although neither linear nor nonlinear models were adequate in relating temozolomide and *C*_max_, or AUC_0−*t*,_ values on day 1 to changes in AGAT activity, a simple *E*_max_ model marginally described the relations between decrement in AGAT activity and total temozolomide dose (*R*^2^=0.104), as shown in Figure 3A, while the relation between the percentage decrement in AGAT activity following 7–21 days of treatment and total drug exposure estimated up to the time of PBMC sampling (AUC_0−*t*_ × days) was more appropriately described by a sigmoidal *E*_max_ model (*R*^2^=0.1881), as shown in Figure 3B.

### Recovery of PBMC AGAT activity

**Figure 4:**
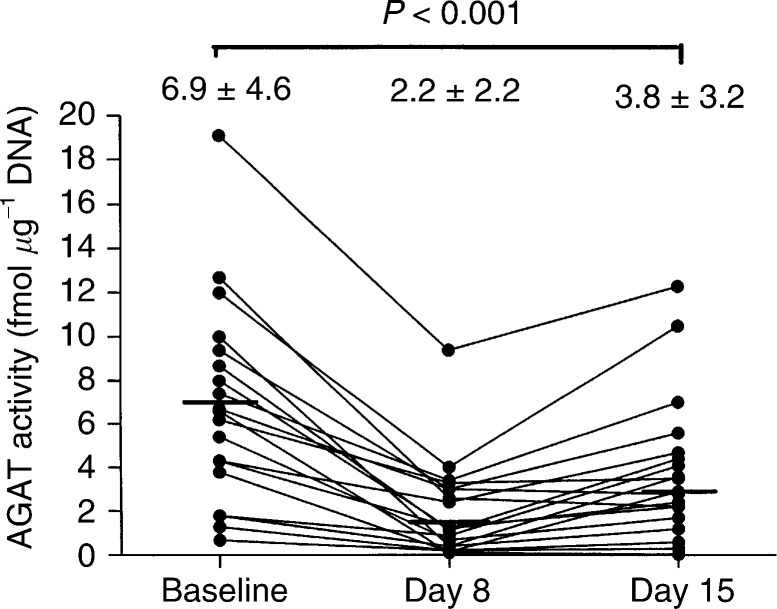
Mean (s.d.) AGAT activity at baseline and day 8, and recovery at day 15 following administration of temozolomide on days 1–8.

The recovery of AGAT activity following treatment was studied thoroughly in patients treated with temozolomide on Schedule A. Marked and significant reductions in AGAT activity from pretreatment levels persisted on day 15 (Figure 4

), which was 7 days after the first series of seven daily treatments and immediately before the next 7-day course (*P*<0.001, paired *t*-test). After the 7-day treatment-free period, mean AGAT activity recovered to only 55% of the initial level prior to treatment.

### *O*^6^-alkylguanine-DNA alkyltransferase inactivation and toxicity

Relations between AGAT inactivation and the principal toxicities of temozolomide on both Schedules A and B were sought. Neither linear nor nonlinear models adequately described the relations between the percentage changes in platelet and neutrophil counts and AGAT inactivation. However, the percentage decrements in AGAT activity were significantly greater in Schedule B patients who experienced grade 3 or 4 thrombocytopenia compared to those who did not experience severe platelet toxicity (mean percentage AGAT inactivation, 90.3±5.5 *vs* 72.5±16.1%, *P*<0.045). Both groups of patients received treatment with similar total temozolomide doses (mean total temozolomide doses, 4515±556 *vs* 4083±1079 mg (*P*=0.44)). The low number of severe thrombocytopenic events in patients treated with temozolomide on Schedule A may have limited the statistical power of similar analyses in these patients.

## DISCUSSION

The results of both experimental and clinical studies have indicated that AGAT activity is an important determinant of tumour sensitivity to several types of DNA-damaging agents, such as methylating and chloroethylating agents, which target the *O*^6^ position of guanine (Dumenco *et al*, 1989; Schold *et al*, 1989; Pegg, 1990; Esteller *et al*, 2000). Therefore, strategies directed at inactivating AGAT, particularly those resulting in preferential AGAT inactivation in malignant tissue, may enhance the antitumour activity, and possibly the therapeutic indices of relevant anticancer agents. The current study was performed to characterise the magnitude and temporal aspects of AGAT inactivation in PBMCs in the course of developing protracted schedules of the oral, DNA methylating agent temozolomide, which has undergone regulatory approval for treating patients with anaplastic astrocytoma on a daily × 5-day every 4-week schedule, and has also demonstrated prominent activity against other central nervous system malignancies and melanoma. The rationale for the present studies is the high rate of anticancer activity noted following treatment of patients with malignant brain tumours with more protracted, low-dose temozolomide schedules (Newlands *et al*, 1992; Brock *et al*, 1998), as well as the hypothesis that the administration of DNA methylating and chlorethylating agents on protracted schedules may progressively lead to greater inactivation of AGAT and inhibition of recovery of AGAT activity to a much greater extent than less protracted schedules. In essence, administration schedules that lead to the progressive inactivation of AGAT may ‘auto-enhance’ the innate cytotoxicity of DNA-damaging agents.

The results of the current study support the assertion of Brock *et al* (1998) that cumulative ‘schedule-dependent’ inactivation of AGAT is, in part, responsible for the level of activity noted in preliminary studies of temozolomide administered on protracted schedules. In the present study, profound and persistent AGAT inactivation was noted with two low-dose protracted schedules of temozolomide. The total dose of temozolomide, duration of treatment and temozolomide exposure (AUC_0−*t*_ × number of days) however were only approximate determinants of AGAT inactivation. Instead, substantial, albeit nearly equivalent, degrees of AGAT inactivation were observed with many iterations of temozolomide doses and protracted treatment periods. These results suggest that there may be thresholds with regard to temozolomide dose levels, treatment periods, and pharmacologic exposure, above which further AGAT inactivation does not occur and with low dose administered for prolonged schedules this threshold is exceeded with temozolomide. For example, although the magnitude of AGAT inactivation appeared to be greater at the highest dose levels following 7 days of treatment, dose-dependent differences in AGAT inactivation were not apparent following 14 and 21 days of treatment. In fact, the MTD for Schedule B, 85 mg m^−2^ day^−1^, resulted in nearly equivalent AGAT inactivation following 14 and 21 days of temozolomide as 7 days of treatment with higher doses (125–175 mg m^−2^ day^−1^). However, these results do not necessarily imply that the antitumour activity of low doses of temozolomide administered for more protracted periods will be equivalent to that resulting from higher doses administered for less protracted periods, since AGAT inactivation may be only one of many, as of yet unknown, determinants of temozolomide activity.

Other investigators have described the absence of a strong relations between temozolomide dose and AUC and AGAT inactivation. Similar to the current study, Gander *et al* (1999) found that no relation was apparent between AGAT inactivation and temozolomide AUC when temozolomide was administered in divided doses over 2 days.

As expected, myelosuppression, particularly severe thrombo-cytopenia, was the principal dose-limiting toxicity of temozolomide in patients treated with the agent on both Schedules A and B. For patients treated on Schedule B, AGAT inactivation in PBMC was significantly greater in patients who experienced severe thrombocytopenia than in those who did not. This relation was apparent despite the heterogeneity of the patient population with regard to the extent of prior therapy, which had previously been demonstrated to be a strong determinant of severe thrombocytopenia on protracted temozolomide schedules (Denis *et al*, 2000; Figueroa *et al*, 2000). Based on the nature of the clinical toxicities observed, as well as experimental evidence indicating that haematopoietic progenitors, particularly megakaryocyte precursors, have inherently low AGAT activity, it may be speculated that these progenitor cells are especially sensitive to strategies that inactivate AGAT (Gerson *et al*, 1996).

Several therapeutic approaches designed to inactive AGAT using methylating agents have been evaluated. One approach to achieve maximal inactivation of AGAT is to administer sequentially DNA-damaging agents; however, initial evaluations using streptozotocin have provided little enthusiasm for broader investigations of this strategy (Willson *et al*, 1995; Smith *et al*, 1996). For example, Willson *et al* (1995) evaluated a sequential regimen of streptozotocin, which was administered to inactivate AGAT, followed by treatment with BCNU. Although this particular approach was supported by preclinical data, in which the drug combination produced superior anticancer activity compared to BCNU alone, improved antitumour activity was not observed, and pulmonary toxicity was substantial (Panella *et al*, 1992; Smith *et al*, 1996). An alternate strategy using imidazotetrazinone methylating agents has been used. Lee *et al* (1993a,1993b) examined the use of sequential DTIC prior to fotemustine. DTIC markedly reduced PBMC AGAT and this effect occurred rapidly (within 1–6 h). The magnitude of AGAT reduction was greater for doses over 400 mg m^−2^ compared to 400 mg m^−2^, but no dose–response effect was noted between 500 and 800 mg m^−2^. Similar to the current study, DTIC also led to prolonged AGAT inactivation. Moreover, Philip *et al* (1996) noted inactivation (that persisted for up to 24 h in 11 patients and AGAT remained partially inactivated after 3 weeks in 13 patients. Other investigators have documented similar reductions of AGAT that only partially recovered 3 weeks from dosing (Middleton *et al*, 2000). These findings are in agreement with the current study, in which AGAT remained partially inactivated after 7 days without temozolomide treatment, and indicate a similarity in time course and persistence of AGAT depletion across the class of imidazotetrazinone methylating agents (Lee *et al*, 1994).

*O*^6^-alkylguanine-DNA alkyltransferase activity in PBMCs has been used as a pharmacodynamic surrogate end point in several clinical studies of DNA-damaging agents, although changes in AGAT activity in PBMCs may not reflect changes in tumour tissues with some agents (Willson *et al*, 1995; Spiro *et al*, 1999). Spiro *et al* (1999) reported that AGAT inactivation in PBMCs occurs at an earlier time point and is more profound in PBMCs than in malignant tissues following treatment with *O*^6^BG. The investigators demonstrated that AGAT activity was undetectable in the PBMCs of patients within 15 min following *O*^6^BG treatment, whereas AGAT inactivation in tumour samples was first noted at least 18 h after treatment (Spiro *et al*, 1999). Furthermore, 5- to 12-fold higher doses of *O*^6^BG were required to inactivate completely intratumoural AGAT (Spiro *et al*, 1999). Other investigators have noted tumour AGAT reductions following temozolomide administration, but these studies were small and the sampling time for AGAT inactivation after temozolomide administration was brief (4 h) (Gander *et al*, 1999). These earlier publications therefore question the accuracy of PBMCs as a surrogate for tumour AGAT activity. It should not be presumed, however, that these results are readily applied to the findings in the current study. The current study's temozolomide schedules used protracted and cumulative exposure to deplete AGAT, and the PBMCs were sampled at later time points to examine these cumulative effects. In contrast, the previous clinical studies of *O*^6^BG and temozolomide utilised schedules that permitted only a relatively brief exposure to *O*^6^BG and sampling of PBMC and tumour tissue AGAT was performed at early time points. The differences between decrements in PBMC and tumour tissue AGAT following a relatively brief exposure to *O*^6^BG and temozolomide may merely reflect the difference between *O*^6^BG plasma and tissue concentrations. In the current study, however, temozolomide dose and plasma concentration (*C*_max_ and AUC_(0−*t*)_) appeared to influence AGAT inactivation only with the short 7-day schedule and not with more prolonged treatment where maximal inactivation occurred even with low doses and plasma concentrations. Since studies have not examined AGAT depletion in PBMC and tumour tissues following protracted *O*^6^BG or temozolomide schedules, it remains to be determined whether the aforementioned pharmacodynamic studies of *O*^6^BG and temozolomide that utilised brief exposure schedules are relevant to the current study utilising protracted schedules. To better address this question, an examination of AGAT activity in PBMC and tumour tissue following prolonged temozolomide exposures would be an appropriate next step in the clinical development of these schedules.

Complete inactivation of AGAT may not be necessary to achieve maximal clinical activity. Instead, there may be specific threshold levels of AGAT activity, below which maximal cytotoxicity occurs (Wedge *et al*, 1997; Kokkinakis *et al*, 2001). This phenomenon has been demonstrated in mice bearing human glioblastoma xenografts, in that progressively decreasing AGAT activity in a tumour cell line with AGAT activity of 45 fmol mg^−1^ protein by administering *O*^6^BG does not result in any further enhancement of alkylator antitumour efficacy (Kokkinakis *et al*, 2001). In addition, both failure-free survival and overall survival were significantly longer following BCNU treatment in patients with brain tumours that expressed low to intermediate levels of AGAT (<60 000 molecules per nucleus) compared to those with tumours expressing high levels of AGAT (progression-free survival, 6 *vs* 3 months (*P*=0.008); overall survival, 29 *vs* 8 months (*P*<0.0002)) (Jaeckle *et al*, 1998). This relation of AGAT expression and clinical outcome in brain tumour patients, a tumour site in which methylating/alkylating agents have some utility, may not be applicable in other malignancies, where the mechanisms of chemotherapy resistance may be independent of AGAT expression (Middleton *et al*, 1998). In the present study, AGAT activity decreased by 80 and 75% following 7 and 21 days of treatment with temozolomide on well-tolerated dose-schedules. However, additional biochemical and clinical studies designed to define a range or threshold level of AGAT inactivation, for which to target with modulating strategies, will undoubtedly facilitate the development of temozolomide dose-schedules with superior therapeutic indices in those tumour sites, where methylating agents have utility (Friedman *et al*, 1998).

The sustained inactivation of AGAT 7 days after treatment with temozolomide on the protracted schedules evaluated in the present study may have important implications for selecting dose-schedules for further clinical evaluations. Although the magnitude of AGAT inactivation and the temporal nature of its regeneration following treatment with temozolomide on its approved schedule, 150–200 mg m^−2^ day^−1^ for 5 days every 4 weeks, have not been studied, it can reasonably be speculated that partial or complete AGAT regeneration would likely occur in the relatively long 23-day interval between each 5-day course of temozolomide. In contrast, both Schedules A and B in the present study incorporate a much shorter 7-day interval between periods of drug treatment, and, therefore, temozolomide is always readministered when AGAT is in an inactivated state. From both biochemical and pharmacological perspectives, Schedule A is particularly interesting. At the recommended dose of temozolomide on Schedule A, 150 mg m^−2^ day^−1^ × 7 days every 2 weeks, peak plasma temozolomide concentrations and AUC_0−*t*_ values would be expected to approach maximally tolerated values, and AGAT inactivation is rapid, marked, and sustained. Furthermore, temozolomide dose intensity on Schedule A is 2.8- to 2.1-fold higher than that achieved with its approved dose-schedule (2100 *vs* 750–1000 mg m^−2^ per 4 weeks). These potential advantages have provided the impetus to begin disease-directed clinical evaluations of temozolomide on Schedule A to determine if superior antitumour activity and/or a broader antitumour spectrum can be achieved (Spiro *et al*, 2001).
